# Residual Tau-Fluvalinate in Honey Bee Colonies Is Coupled with Evidence for Selection for *Varroa destructor* Resistance to Pyrethroids

**DOI:** 10.3390/insects12080731

**Published:** 2021-08-14

**Authors:** María Benito-Murcia, Carolina Bartolomé, Xulio Maside, José Bernal, José Luis Bernal, María Jesús del Nozal, Aránzazu Meana, Cristina Botías, Raquel Martín-Hernández, Mariano Higes

**Affiliations:** 1 Centro de Investigación Apícola y Agroambiental (CIAPA), Laboratorio de Patología Apícola, Instituto Regional de Investigación y Desarrollo Agroalimentario y Forestal (IRIAF), 19180 Marchamalo, Spain; mbmurcia@jccm.es (M.B.-M.); cbotias@jccm.es (C.B.); rmhernandez@jccm.es (R.M.-H.); 2 Grupo de Medicina Xenómica, CIMUS (Instituto de Investigación de Santiago De Compostela), Universidad de Santiago de Compostela, 15782 Santiago de Compostela, Spain; carolina.bartolome@usc.es (C.B.); xulio.maside@usc.es (X.M.); 3 Instituto de Investigación Sanitaria de Santiago (IDIS), 15706 Santiago de Compostela, Spain; 4 I.U. CINQUIMA, Analytical Chemistry Group, Faculty of Sciences, University of Valladolid, 47002 Valladolid, Spain; jose.bernal@uva.es (J.B.); joseluis.bernal@uva.es (J.L.B.); mjdnozal@qa.uva.es (M.J.d.N.); 5 Department of Animal Health, Faculty of Veterinary Medicine, Complutense University of Madrid, 28040 Madrid, Spain; ameana@ucm.es; 6 Instituto de Recursos Humanos para la Ciencia y la Tecnología (INCRECYT-FEDER), Fundación Parque Científico y Tecnológico de Castilla-La Mancha, 02006 Albacete, Spain

**Keywords:** *Varroa destructor*, mite, resistant populations, acaricide residues, tau-fluvalinate, varroosis

## Abstract

**Simple Summary:**

*Varroa destructor* is one of the most prevalent honey bee (*Apis mellifera*) pathogens worldwide. Nowadays, the main method to control this parasite involves the application of different acaricidal treatments, among which the pyrethroid tau-fluvalinate is one of the most widely used. However, the intensive and repetitive application of these chemicals generates a selective pressure that, when maintained over time, contributes to the emergence of resistant mites in the honey bee colonies. Here we analysed the presence of residual tau-fluvalinate and the patterns of genetic resistance to this acaricide in *Varroa* mites collected from tau-fluvalinate untreated honey bee colonies. Our results show the widespread and persistent pyrethroid contamination of beeswax and beebread in the hives, along with an excess of pyrethroid-resistant genotypes and an overall increase in the frequency of the pyrethroid-resistant allele in the mite population over time. Persistent contamination of the hives likely compromises the efficacy of tau-fluvalinate treatments and, therefore, may have serious long-term consequences for the control of varroosis.

**Abstract:**

*Varroa destructor* is considered one of the most devastating parasites of the honey bee, *Apis mellifera*, and a major problem for the beekeeping industry. Currently, the main method to control *Varroa* mites is the application of drugs that contain different acaricides as active ingredients. The pyrethroid tau-fluvalinate is one of the acaricides most widely used in beekeeping due to its efficacy and low toxicity to bees. However, the intensive and repetitive application of this compound produces a selective pressure that, when maintained over time, contributes to the emergence of resistant mites in the honey bee colonies, compromising the acaricidal treatments efficacy. Here we studied the presence of tau-fluvalinate residues in hives and the evolution of genetic resistance to this acaricide in *Varroa* mites from honey bee colonies that received no pyrethroid treatment in the previous four years. Our data revealed the widespread and persistent tau-fluvalinate contamination of beeswax and beebread in hives, an overall increase of the pyrethroid resistance allele frequency and a generalized excess of resistant mites relative to Hardy–Weinberg equilibrium expectations. These results suggest that tau-fluvalinate contamination in the hives may seriously compromise the efficacy of pyrethroid-based mite control methods.

## 1. Introduction

The ectoparasitic honey bee mite *Varroa destructor* (Acari: Varroidae, Anderson and Trueman, 2000) is widespread in *Apis mellifera* (Hymenoptera: Apidae, Linnaeus, 1758) colonies worldwide after it shifted from its original host, the Eastern honey bee *Apis cerana* (Hymenoptera: Apidae, Fabricius, 1793) [1]. It is considered a major problem for beekeeping [2], with the potential to affect individual bees and whole honey bee colonies, and if not treated adequately it can ultimately lead to colony loss within 2–3 years [1]. Indeed, several factors contribute to the dramatic effect of *Varroa* infection on honey bee populations, including a direct effect on the feeding of immature and adult bees, as well as serving as a vector for several debilitating viruses [1,3,4,5,6].

Currently, the main method to control *Varroa* mites is the application of veterinary drugs based on different compounds with acaricide activity. However, after several decades using these compounds (mainly pyrethroids and organophosphates) a loss of their efficacy is a reality in many countries, as intensive and repetitive use may exert a selective pressure that favours the emergence of resistant mite populations [7,8,9,10,11,12,13]. Tau-fluvalinate is one of the most widely used acaricides in beekeeping due to its efficacy and low bee toxicity [14]. Thus, intensive and repetitive application of this drug is associated with the detection of resistant mite populations, first in Italy in the 1990s and subsequently elsewhere [15,16]. Currently in Spain, and due to doubts about the field efficacy of fluvalinate [12] the use of veterinary drugs with this acaricide has decreased (approximately 15% f bee colonies are treated) while the use of amitraz has increased (applies to 80% of colonies).

Tau-fluvalinate is a pyrethroid that targets voltage-gated sodium channels (*VGSCs*), disrupting their activity in neural signalling and producing paralysis in affected arthropods. Pyrethroid resistance, especially extreme resistance known as knockdown resistance (*kdr*), has been attributed to mutations of key residues in *VGSCs* [17]. Indeed, several point mutations in the III and IV domains of *VGSC* genes have been identified in resistant *Varroa* mites [18]. More recently, several amino acid substitutions were also described in domain II at position 925 (numbered according to *Musca domestica* Linnaeus (Diptera: Muscidae)), lying in segment 5 of the *VGSC* helix that is the region in which a hydrophobic cavity is formed for pyrethroid binding [19]. As such, three different resistant alleles have been described at this position, replacing wild-type leucine with valine (L925V), isoleucine (L925I) or methionine (L925M) [10,20,21,22,23,24,25] Accordingly, the L925V in *V. destructor* appears to be a key change in *kdr* and the presence of populations carrying these mutations may be favoured when acaricide treatments containing tau-fluvalinate are maintained over time.

Due to the high levels of miticides and agrochemicals found in wax and pollen around the world and given that these are mainly tau-fluvalinate [26,27,28,29,30,31,32,33], we set out to investigate the possible relationship between such residues in wax and beebread, and their interaction with *Varroa* mite populations. In this way, our aim was to better understand whether the persistence of such residues might be responsible for favouring the emergence of *Varroa*-resistant mites, since beekeepers frequently report problems in controlling varroosis with these acaricide after years of not using it.

## 2. Materials and Methods

### 2.1. Honey Bee Colonies Selection and Location

This study was carried out from October 2018 to October 2019 on 10 honey bee colonies (A11–A20; Table 1 and Table S1) that displayed natural parasitization by *V. destructor* and that had not received any acaricide treatment containing pyrethroids as active ingredient (Apistán^®^, active substance: tau-fluvalinate. Bayvarol^®^, active substance: flumethrin) in the previous 48 months (since October 2014). During this time they were treated once a year (autumn) with amitraz and oxalic acid alternately. The bee colonies were located in 2014 in an experimental apiary at CIAPA (Marchamalo, central Spain, LT: 40.687756-LG: −3.218516), separated more than 5 km from other apiaries that could alter the results of the study.

### 2.2. Wax and Beebread Samples

#### 2.2.1. Sample Collection

Beeswax and beebread samples were taken from each of the hives. These were subject to a preliminary multi-residue analysis (modified QuEChERS method) to assess the potential presence of more than 200 chemicals in these matrices. Their recovery was performed by using graphitized carbon black and primary-secondary amine in combination with magnesium sulfate in a cartridge, which was eluted with a mixture of acetone and toluene. The resulting extracts were analyzed by gas chromatography-mass spectrometry (GC/MS) [27] that revealed that the only pyrethroid present in these matrices was tau-fluvalinate.

The detection of this chemical in most beeswax samples, even in those theoretically decontaminated [27,34,35,36], prevented the possibility of setting a reliable negative control (colonies containing fluvalinate-free combs and supplemented with uncontaminated pollen). On the other hand, the inclusion of a positive control (colonies receiving applications of tau-fluvalinate) was discarded because honey bee drifting could alter the genotypic/allelic frequencies of the mites infecting untreated colonies.

Wax and beebread samples (n = 24 of each type) were collected at different time points (autumn 2018, spring 2019 and autumn 2019, Figure 1). For wax sampling, brood chamber combs (empty and with beebread) were taken from each hive and then, 4 pieces of wax (4 × 4 cm) were collected from different parts of the combs. Beebread (10 g) was taken randomly from different cells of combs with this matrix, using sterile stainless-steel spatulas. All the samples were kept at −20 °C.

#### 2.2.2. Chemical Analysis

The wax and beebread samples were sent on dry ice to the Analytical Chemistry Group of the University of Valladolid (I.U. CINQUIMA, Valladolid, Spain) for analysis.

The monitoring of tau-fluvalinate was carried out in a GC-MS system using a method described previously [37] based in solvent extraction with an ethyl acetate and hexane (70:30, *v*/*v*) mixture. Then, the resulting solution was vortexed vigorously for 20 min and centrifuged for 10 min. Afterwards, 6 mL of the supernatant were transferred to 10 mL a centrifuge tube containing 500 mg of Z-sep/C_18_ sorbent. Samples were again vortexed and centrifuged employing the aforementioned conditions. Finally, a 3 mL aliquot of the extract was evaporated to dryness, and the residue was re-dissolved in 0.5 mL of 0.1% acetic acid in hexane prior to a GC/MS analysis on an Agilent 6850 GC with a 5975C MS detector, which was used in negative chemical ionization mode. The analytes were separated using a HP-5MS (30 m × 0.25 mm × 0.25 µm) column. Extraction was firstly carried out by a modified version of the QuEChERS method [26,38] In this specific study, and taking into account the possible difference in the expected final concentrations, we selected a recently commercialized sorbent (EMR-lipid) with the aim of removing this family of compounds, which are quite usual in this matrix. This modification of the QuEChERS method allowed cleaner chromatograms to be obtained and an improvement of the reproducibility in a shorter time. Briefly, 2 g of wax or 0.5 g of pollen were added to a 50 mL polypropylene centrifuge tube containing 10 mL of acetonitrile–acetic acid (99:1, *v*/*v*), mixed by vortexing for 15 s and then for 3 min with an Ultra Turrax homogenizer. The samples were centrifuged at 10,000 rpm for 5 min at 5 °C, and 5 mL of the extract was added to a dSPE EMR-lipid cartridge (Agilent), vortexed for 30 s and centrifuged for 5 min at 7500 rpm and 5 °C. The supernatant (5 mL) was then pipetted into a 15 mL centrifuge tube containing the “Final Polish EMR-lipid” sorbent (Agilent), vortexed for 30 s and then centrifuged for 5 min at 7500 rpm and 5 °C. Subsequently, 2 mL of the supernatant were placed in a round bottom flask, evaporated to dryness in a rotary evaporator and re-dissolved in 1 mL of an ethyl acetate-cyclohexane mixture (20:80, *v*/*v*) in an ultrasound bath. The sample was then analysed on an Agilent GC with a 5975-quadruple mass detector using chlorfenvinphos-D10 as the internal standard.

### 2.3. Varroa Mite Collection and Genotyping

#### 2.3.1. Mite Collection

**Figure 1:**
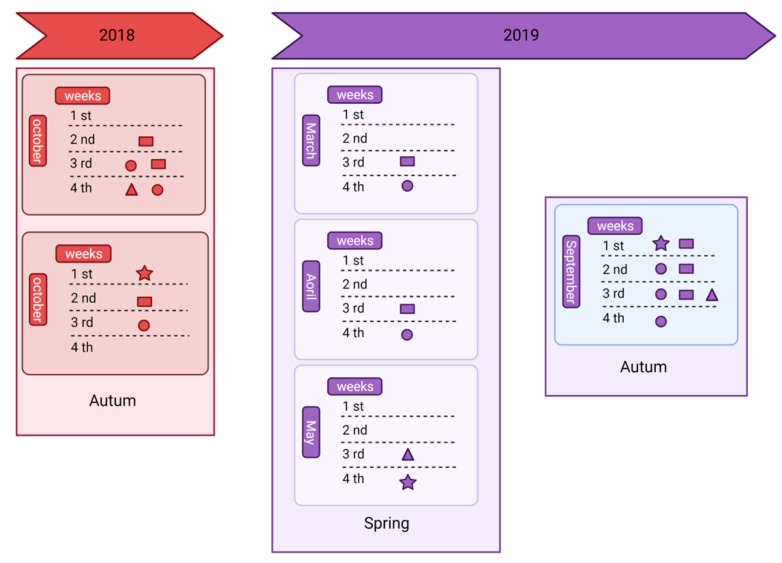
Chronology of the operations performed in the colonies. Rectangle: put clean sanitary bottom. Circle: remove sanitary bottom. Star: treatment against *V. destructor* with amitraz. Triangle: wax and beebread sampling. Created by Biorender software.

Adult female *Varroa* mites were sampled by placing a tray at the sanitary bottom of each hive [38] that was checked once a week. The collection was performed at nine time points between the autumns of 2018 and 2019 (Figure 1 and Table S1); these included samples taken before and after the application of mandatory acaricide treatments (Figure 1; Apitraz^®^ strips: 2 strips/hive, active ingredient: 500 mg of amitraz per strip), as indicated in the Spanish legislation on the control of varroosis [39]. The objective behind this strategy was to determine if the application of amitraz had any effect on the mortality of pyrethroid-resistant mites. Specimens were then stored at −20 °C for later DNA extraction.

#### 2.3.2. DNA Extraction and PCR-RLFP Assay

Individual mites were ground in separate wells in a 96-well plate (Qiagen, Hilden, Germany), to which 200 µL of sterile H_2_O miliQ and three stainless steel beads were added, shaking the plates for 1.5 min in a TissueLyser II machine at 30 Hz (Qiagen, Cat. No./ID: 85300). Subsequently, 150 µL of the macerate were incubated overnight at 56 °C with 20 µL of Proteinase K (Qiagen, Cat No./ID: 19133) as described elsewhere [40,41]. Genomic DNA was extracted in a Biosprint workstation (Qiagen) using the BioSprint 96 DNA Blood Kit (Qiagen, Cat No./ID: 940057)—following the BS96 DNA Tissue protocol—and stored at −20 °C until use. Negative controls were included for each step of the process.

The genotype of the mites at position 925 (domain II) of the VGSC gene was determined by applying a PCR-RFLP protocol described previously [41,42]. Briefly, the PCR reaction mixtures were performed with 0.5 µM of each oligonucleotide primer (forward primer [1273-IF_VD] 5′-AAGCCGCCATTGTTACCAGA-3′; reverse primer [1973-IR_VD] 5′-CTGTTGTTACCGTGGAGCA-3′), 0.5 µL of BSA + Triton X-100, and 2.5 µL genomic DNA and 12.5 µL of the Fast Start PCR Master mix (Cat No. 04710452001 Roche Diagnostic, Indianapolis, IN, USA) in a final volume of 25 µL. The cycling conditions were: 10 min at 95 °C; 35 cycles of 30 s at 95 °C, 30 s at 60 °C, 45 s at 72 °C; and a final extension for 7 min at 72 °C. All reactions were carried out in a Mastercycler ep gradient S (Eppendorf, Hamburg, Germany) and analysed in a QIAxcel Advanced System (Qiagen) using a QIAxcel DNA Screening Kit to confirm amplification (Qiagen, Cat No. 929004). Subsequently, 15 μL of each PCR products were mixed with 2.5 U of SacI (Thermofisher Scientific, Waltham, MA, USA, Cat No: #ER1131) and 3 μL of 10× SacI buffer in a final volume of 20 μL, and the products were digested for 4 to 16 h at 37 °C. This digestion should generate two fragments (264 bp and 436 bp) from the wild-type genotype and a single 700 bp ‘undigested’ fragment in the case of the resistant strains. Finally, the products of digestion were analysed by capillary electrophoresis and visualized with a QIAxcel Advanced System (Qiagen).

To ascertain the correct genotyping classification of mites with the PCR-RLFP method used, 75 DNA samples were genotyped by Sanger sequencing. The samples were selected according to the genotypes’ relative abundances: 8 resistant homozygotes, 65 sensitive homozygotes and 2 heterozygotes. DNA extracts of the samples were subject to a previously described PCR protocol [22] that amplifies a 170 bp fragment of the *VGSC* gene encompassing ‘hot spot’ resistance positions—including position 925-. Subsequently PCR products were purified with QIAquick PCR Purification Kit (Qiagen, Cat No./ID: 28106) and sequenced at the Universidad de Alcalá de Henares (Madrid, Spain) on an ABI3730XL Applied Biosystems system. The sequences obtained were analysed with BioEdit (BioEdit sequence Aligment Editor^©^ version 7.2.5), which allowed us to identify the different *Varroa* genotypes.

### 2.4. Allele Frequencies

At each sampling date the allele frequencies of the sensitive (S) and resistant (R) alleles, *p* and *q* respectively, in each colony were estimated from the observed PCR-RFLP genotypes:

*p* = (2SS + SR)/2*N*

*q* = 1 − *p*

where SS is the number of individuals homozygous for the sensitive allele, SR, the number of heterozygous, RR, the number homozygous for the resistance allele, and *N* the total number of specimens analysed.

The expected genotype frequencies assumed under Hardy–Weinberg equilibrium were estimated using the usual formulae:

SS = *p^2^ N*; SR = (2*pq*)*N*; RR = *q^2^N*

The deviation of the genotype frequencies observed from those expected under equilibrium were evaluated using a Chi-squared goodness of fit test.

To obtain more accurate estimates of the observed genotype frequencies, data were pooled into three periods of about one month each (autumn 2018: 15 October 2018 to 19 November 2018, spring 2019: 26 March 2019 to 29 April 2019 and autumn 2019: 4 September 2019 to 25 September 2019, respectively) representing different generations of *Varroa* mites. Before doing so, the differences among the observed genotype frequencies across colonies of each given sampling date were evaluated by means of the Chi-square homogeneity test. The Bonferroni correction for multiple testing and the Yates’ correction for continuity were applied when appropriate. No statistically significant differences were observed on all but two sampling dates: 23 October 2018 and 17 September 2019 (Table S1). In these cases the differences could be attributed to comparatively higher frequencies of heterozygotes in two (A19, A20) and one (A15) colonies, respectively. However, considering that the three colonies produced similar results to the others in the immediately anterior and posterior sampling dates, which were performed in 8-day intervals (Table S1), the data from these samples were not removed from the analyses. In the pooled colony data, heterogeneity among colonies was detected only in autumn 2018, again as a result of the odd values observed in colonies A19 and A20. 

## 3. Results

### 3.1. Tau-Fluvalinate Residues in Wax and Beebread

The preliminary multi-residue analysis of the colonies showed that tau-fluvalinate was present in all the samples in significant quantities and to a lesser extent and frequency: coumaphos, chlorfenvinphos and chlorpyrifos. In addition, some residues of atrazine, α-endosulfan and bromopropilate were detected in a few cases. 

Subsequent analyses proved that tau-fluvalinate was present throughout the study both in wax and beebread (Table 1). The highest concentrations were detected in the beebread, with values ranging from 1960.5 ± 76.2 ppb (mean ± standard deviation) in autumn 2018 (range 1863.3 to 2129.3 ppb) to a lower level of 674.4 ± 128.7 ppb in spring 2019 (range 450.1–901.8 ppb), before falling to 303.7 ± 139.2 in autumn 2019 (range 167.1–541.2 ppb). The values of the residues detected in the wax of the brood chamber combs were constant over time, with average concentrations ranging from 70.4 ± 25.0 ppb in autumn 2018 to 73.2 ± 16.2 ppb in spring 2019 and 68.6 ± 11.7 in autumn 2019 (Table 1).

### 3.2. Pyrethroid-Resistance Allele Frequencies

A total of 1516 *V. destructor* female mites were sampled from 10 honey bee colonies at nine different time points in 2018 and 2019. The genotype at position 925 (domain II) of the VGSC gene was determined by means of the PCR-RFLP technique (Table 2). The genotype of 75 of these mites was double-checked by Sanger sequencing of the PCR products, with identical results.

Genotype frequencies were obtained by pooling the observed frequencies across colonies at each sampling period (Table 2). In all cases the genotype frequencies departed significantly from Hardy–Weinberg equilibrium expectations (HWE). This effect could be attributed to an overall excess of mites homozygous for the resistance allele (RR), which were 5.3 ± 1.63 (mean ± SE) times more abundant than expected and a concomitant dearth of SR heterozygotes (0.6 ± 0.05). Contrastingly, no departure form HWE expectations was detected for sensitive homozygotes (SS; 1.0 ± 0.01). The two treatments with amitraz had not detectable effects on the frequencies of RR individuals, as expected considering that this miticide does not target pyrethroid receptors [43].

**Figure 2:**
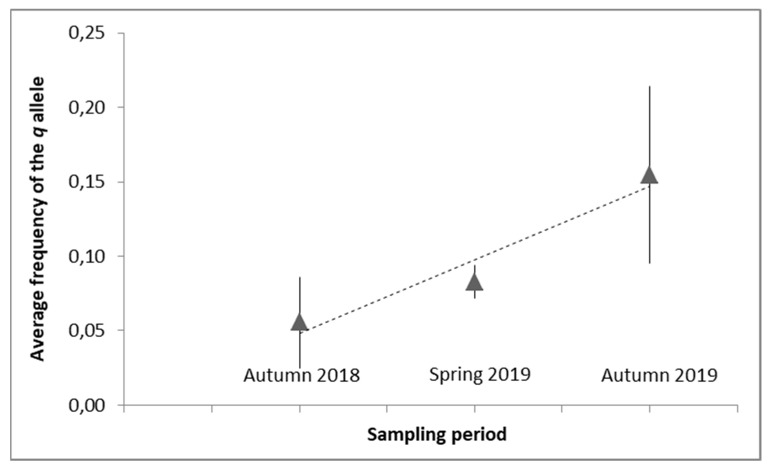
Observed frequency of the resistance allele (*q*) over time (average *q* across pooled samples). The error bars indicate the 95% confidence intervals.

It should also be noted that the frequency of the resistance allele (*q*) experienced a gradual increase over time, from an average of 0.06 ± 0.03 in autumn 2018 to 0.15 ± 0.06 in autumn 2019 (from data in Figure 2). This increment was consistent and independent of the number of samples analysed in each period: *N* = 818 in autumn 2018, *N* = 86 in spring 2019 and *N* = 612 in autumn 2019, respectively.

The analysis of the mean frequency of *q* in the five colonies that were alive at the end of the study revealed a tendency to increase in parallel with the levels of tau-fluvalinate in wax (significant for colonies A12 and A19; Table 3), and vice versa (significant for colony A20 too). This trend was not found in the case of colonies A15 and A16, in which the frequency of *q* increased independently of the levels of acaricide. The former showed similar concentrations of tau-fluvalinate at the beginning and at the end of the experiment, whereas A16 displayed lower levels in 2019 than in the previous year; however, it must be noted that this colony exhibited the highest concentration of fluvalinate (as well as the highest mean frequency of *q*) in 2018, which could have been enough to boost the frequency of the resistance allele despite the reduction in the levels of pyrethroid in wax during the following year.

## 4. Discussion

A widespread and persistent tau-fluvalinate contamination of beeswax and beebread was detected in the hives studied despite the fact that these were not treated with this acaricide in the previous 48 months. The highest concentrations were found in beebread, probably due to the active migration by diffusion/partition from the beeswax to the stored pollen [40]. Beeswax is a lipophilic matrix that can retain non-polar pesticides, which can, therefore, be transferred and distributed to other hive products [43,44,45]. Thus, higher affinity for lipophilic miticides of beebread compared to wax underlies the concentration of tau-fluvalinate in this matrix [43].

However, these higher concentrations may also be due to the presence of these residues in the pollen collected by bees from crop plants treated with tau-fluvalinate, although the limited use of this active ingredient on crops in the study area (according to official data [46]) does not support this hypothesis. Moreover, the decrease in the average concentration of tau-fluvalinate detected in the beebread collected in spring as opposed to autumn probably reflects a buffering effect due to the input of fresh uncontaminated pollen in spring [45] when the foraging activity of honey bees is at its peak.

The average levels of tau-fluvalinate detected in beebread samples are lower than the LC50 values reported for honey bee larvae (27.69 ppm: [47]) and the concentrations considered to cause high mortality in 1 day-old larvae (3 ppm: [48]). Nevertheless, the presence of tau-fluvalinate residues in all beebread and wax samples is of concern, since the persistence of this pyrethroid in a honey bee colony may have a potentially negative effect on the weight, oviposition and survival of queens, as well as on the sexual competitiveness and survival of drones [49,50,51]. 

Another detrimental effect of the continuous exposure of honey bee colonies to this chemical may be the selection for resistance in the *Varroa* mite populations and thus, the loss of tau-fluvalinate efficacy to control varroosis. Indeed, *Varroa* mites are likely to be exposed to residual tau-fluvalinate directly from the wax, but also from the fat body of honey bees contaminated with these residues (lipophilic pesticides like tau-fluvalinate have been proven to accumulate in the fat body of bees [41,42,43,44,45,46,47,48,49,50,51,52,53] and *Varroa* mites feed primarily on this tissue [4]). 

The extensive finding of pyrethroid residues in beeswax [27,34] and the long-lasting contamination they produce on hive matrices [35], are likely to drive the persistence of resistance alleles in *Varroa* populations [12], despite their high fitness cost (that should make them segregate at increasingly lower frequencies in the absence of these acaricides [21]).

The colonies under study were infected naturally when they were introduced in the apiary four years earlier (2014). This means that the resistance allele detected since the beginning of the 2018–2019 survey was probably present in the original *Varroa* population at an initial frequency derived from the genotypes of the mites that survived previous non-pyrethroid treatments (amitraz and oxalic acid). At any rate, the overall excess of resistant genotypes (RR) observed is consistent with selection for pyrethroid resistance in the mite population [53,54,55,56,57].

The analysis of the allele frequencies exhibited a gradual increase in the frequency of the resistance allele (R) over time that is difficult to reconcile with the exposure of these mites to rather constant concentrations of tau fluvalinate in wax (means of 70.4, 73.2 and 68.6 ppb for each of the periods studied). However, when colonies were analysed individually, the increment in the levels of tau fluvalinate in wax tended to correlate with an increase in *q* (and vice versa). In a few cases this pattern was not observed and therefore, in absence of a negative control based on perfectly clean hive materials and food supplements (which, on the other hand, are nearly impossible to obtain [34,36]), we cannot prove the hypothesis that both parameters are directly linked. In any case, the persistence of these alleles in mite population is very likely driven by the ubiquitous presence of pyrethroids in lipophilic materials, which exert a selective pressure on their survival [21].

The life cycle of the parasite might also influence the frequency of the *VGSC* genotypes. *Varroa* mites are known to undergo frequent full sibling mating in the host brood cells under conditions of low mite density and large brood availability (spring and early summer), whereas higher mite density (autumn and winter) prompts outcrossing and recombination [1,52,53,54,55,56,57]. Indeed, this inbreeding could partially account for the observed dearth of heterozygotes in most samples relative to HWE expectations, but it does not explain why the concomitant surplus of homozygotes was only observed for tau-fluvalinate resistant individuals (RR) and not for sensitive ones (SS), when the latter are known to have greater fitness than the former.

At any rate, seasonal fluctuations in the breeding conditions of the mite population may have practical implications. For instance, it has been proposed that in order to minimize selection for the resistant RR genotypes, tau-fluvalinate treatments should be administered when the largest proportion of resistance alleles are in heterozygous mites, while this treatment should be avoided in the strong inbreeding period (i.e., spring–early summer) [53,57]. However, as deducted from the results in this study, the constant presence of tau-fluvalinate residues in the hive could have counter-effects and render this strategy ineffective. 

## 5. Conclusions

Our data revealed the chronic presence of tau-fluvalinate residues in wax and beebread from honey bee colonies that were not treated with this acaricide in the previous four years. This was coupled with evidence for selection for tau-fluvalinate-resistant *Varroa* mites. These results encourage the use of clean, pesticide-free beeswax and the need to regulate the sources of wax being rendered for resale, thereby mitigating the undesired selective effect that these residues may exert on mite populations.

## Figures and Tables

**Table 1:** Tau-fluvalinate concentration (in ppb) detected in wax and beebread from each honey bee colony included in the study at each sampling point (colony A17 died on March 2019 and was replaced by A11). SD: standard deviation.

Colony	Autumn 18		Spring 19		Autumn 19	
	Wax	Beebread	Wax	Beebread	Wax	Beebread
A11	-	-	90.6	680.1	90.2	167.1
A12	24.4	1863.3	58.4	713.7	57.5	172.3
A13	92.1	1943.6	103.6	660.6	-	-
A14	82.6	1960.7	58.0	704.3	-	-
A15	68.2	1902.9	69.1	715.2	64.0	260.9
A16	107.7	1940.1	72.4	723.1	70.1	340.2
A17	61.3	1929.6	-	-	-	-
A18	70.6	2129.3	80.3	450.1	-	-
A19	44.9	1954.7	54.1	520.4	60.1	340.3
A20	82.0	2020.1	72.5	901.8	69.6	541.2
Mean	70.4	1960.5	73.2	674.4	68.6	303.7
SD	25.0	76.2	16.2	128.7	11.7	139.2

**Table 2:** Genotype frequencies observed (Obs) vs. expected (Exp) under Hardy–Weinberg equilibrium. Pooled data across samplings. SS: homozygotes for the sensitive allele, SR: heterozygotes, and RR: homozygotes for the resistance allele; Obs: observed number of mites; Exp: expected number of mites under Hardy–Weinberg equilibrium; *p*, departure of the observed genotype frequencies from those expected under Hardy–Weinberg equilibrium; ***, *p* < 0.001, as estimated by means of a chi-square test of goodness of fit.

Sampling Period		SS	SR	RR	*p*
Autumn 2018	Obs	747	50	21	
	Exp	728.6	86.8	2.6	***
Spring 2019	Obs	75	8	3	
	Exp	72.6	12.9	0.6	***
Autumn 2019	Obs	459	117	36	
	Exp	437.6	159.8	14.6	***

**Table 3:** Mean frequency (±SE) of the resistance allele (*q*) vs. levels of tau-fluvalinate in wax in the colonies that were alive at the end of the experiment.

Colony	Frequency of *q* (Mean ± SE)		Tau-Fluvalinate in Wax	
	Autumn 2018	Autumn 2019	Autumn 2018	Autumn 2019
A12	0.01 ± 0.01	0.08 ± 0.04	24.4	57.5
A15	0.04 ± 0.01	0.16 ± 0.06	68.2	64.0
A16	0.15 ± 0.04	0.18 ± 0.05	107.7	70.1
A19	0.05 ± 0.05	0.19 ± 0.05	44.9	60.1
A20	0.06 ± 0.05	0.00 ± 0.00	82.0	69.6

## Data Availability

The data presented in this study are available on request from the corresponding author.

## Supplementary Materials

The following are available online at https://www.mdpi.com/article/10.3390/insects12080731/s1, Table S1: Observed genotypes at each sampling date.
