# Supplementary material for: Residual Tau-Fluvalinate in Honey Bee Colonies Is Coupled with Evidence for Selection for Varroa destructor Resistance to Pyrethroids
Source: Insects. 2021 Aug 14;12(8):731. doi: 10.3390/insects12080731 (PMC8397018; doi:10.3390/insects12080731)
Supplement: Supplementary file 1 [file insects-12-00731-s001.zip › insects-1328656-SI.pdf]

Table S1. Observed genotypes at each sampling date (blank cells indicate that the colony has died).

15.10.18

Colony	SS	SR	RR	Total
A12	20	0	0	20
A13	18	1	1	20
A14	28	1	0	29
A15	37	0	1	38
A16	27	2	1	30
A17	19	1	1	21
A18	28	4	0	32
A19	29	0	0	29
A20	36	0	0	36
	242	9	4	255

23.10.18

Colony	SS	SR	RR	Total
A12	44	0	1	45
A13	29	7	2	38
A14	37	0	0	37
A15	39	0	2	41
A16	4	0	1	5
A17	0	0	0	0
A18	33	3	1	37
A19	30	12	0	42
A20	23	12	0	35
	239	34	7	280

19.11.18

Colony	SS	SR	RR	Total
A12	37	0	0	37
A13	24	2	1	27
A14	28	0	0	28
A15	37	0	2	39
A16	23	0	5	28
A17	24	1	2	27
A18	33	2	0	35
A19	38	1	0	39
A20	22	1	0	23
	266	7	10	283

26.03.19

Colony	SS	SR	RR	Total
A11	2	0	0	2
A12	4	0	0	4
A13	1	0	0	1
A14	0	0	0	0
A15	0	0	0	0
A16	15	6	0	21
A17				

A18	0	0	0	0
A19	5	0	0	5
A20	1	0	0	1
	28	6	0	34

29.04.19

Colony	SS	SR	RR	Total
A11	0	0	0	0
A12	0	0	0	0
A13	1	0	0	1
A14	2	0	0	2
A15	3	0	0	3
A16	0	0	0	0
A17				
A18	15	1	2	18
A19	0	0	0	0
A20	26	1	1	28
	47	2	3	52

04.09.19

Colony	SS	SR	RR	Total
A11	17	3	0	20
A12	21	5	0	26
A13				
A14				
A15	38	1	5	44
A16	36	1	2	39
A17				
A18				
A19	5	3	0	8
A20	0	0	0	0
	117	13	7	137

09.09.19

Colony	SS	SR	RR	Total
A11	22	3	4	29
A12	11	0	0	11
A13				
A14				
A15	38	4	0	42
A16	29	5	4	38
A17				
A18				
A19	34	2	4	40
A20	3	0	0	3
	137	14	12	163

17.09.19

Colony	SS	SR	RR	Total
A11	26	5	1	32

A12	29	1	1	31
A13				
A14				
A15	16	27	2	45
A16	24	6	2	32
A17				
A18				
A19	26	3	2	31
A20				
	121	42	8	171

25.09.19

Colony	SS	SR	RR	Total
A11	11	12	1	24
A12	25	6	3	34
A13				
A14				
A15	30	7	2	39
A16	16	19	3	38
A17				
A18				
A19	2	4	0	6
A20				
	84	48	9	141
